# Assessment of a UWB Real Time Location System for Dairy Cows’ Monitoring

**DOI:** 10.3390/s23104873

**Published:** 2023-05-18

**Authors:** Provvidenza Rita D’Urso, Claudia Arcidiacono, Matti Pastell, Giovanni Cascone

**Affiliations:** 1 Department of Agriculture, Food and Environment (Di3A)—Building and Land Engineering Section, University of Catania, Via Santa Sofia n° 100, 95123 Catania, Italy; provvidenza.durso@unict.it (P.R.D.); gcascone@unict.it (G.C.); 2 Natural Resources Institute Finland, Luke Latokartanonkaari 9, 00790 Helsinki, Finland; matti.pastell@luke.fi

**Keywords:** monitoring, error assessment, real time measurement, ultra-wideband technology, laboratory experiment, cow localization

## Abstract

In the field of precision livestock farming, many systems have been developed to identify the position of each cow of the herd individually in a specific environment. Challenges still exist in assessing the adequacy of the available systems to monitor individual animals in specific environments, and in the design of new systems. The main purpose of this research was to evaluate the performance of the SEWIO ultrawide-band (UWB) real time location system for the identification and localisation of cows during their activity in the barn through preliminary analyses in laboratory conditions. The objectives included the quantification of the errors performed by the system in laboratory conditions, and the assessment of the suitability of the system for real time monitoring of cows in dairy barns. The position of static and dynamic points was monitored in different experimental set-ups in the laboratory by the use of six anchors. Then, the errors related to a specific movement of the points were computed and statistical analyses were carried out. In detail, the one-way analysis of variance (ANOVA) was applied in order to assess the equality of the errors for each group of points in relation to their positions or typology, i.e., static or dynamic. In the post-hoc analysis, the errors were separated by Tukey’s honestly significant difference at *p* > 0.05. The results of the research quantify the errors related to a specific movement (i.e., static and dynamic points) and the position of the points (i.e., central area, perimeter of the investigated area). Based on the results, specific information is provided for the installation of the SEWIO in dairy barns as well as the monitoring of the animal behaviour in the resting area and the feeding area of the breeding environment. The SEWIO system could be a valuable support for farmers in herd management and for researchers in the analysis of animal behavioural activities.

## 1. Introduction

The need to monitor large herds housed in livestock systems has enhanced the development of automated monitoring systems. In the field of precision livestock farming (PLF), many systems have been developed to monitor the behaviour of cows in a specific environment, improve animal welfare and ensure food safety for consumers [1].

Based on the literature the main monitoring technologies are the following: image analysis using video recordings [2,3], sound analysis [4], computer vision [5,6], accelerometers [7,8,9], pedometers [10,11,12], Bluetooth technology-based systems [13], radar technology [14], UHF technology-based systems [15]. The use of these monitoring systems could support researchers in the monitoring of animal behavioural activities as well as farmers in herd management. Different information on animal behaviour can be provided: occurrence of cow’s oestrus [8,16], identification preferences of cows in barn areas, early detection of disease (e.g., lameness, mastitis) [17], number of milkings in robotic milking systems, time spent at specific activities (i.e., drinking, lying, perching, feeding, standing, and walking) [18,19,20], the temporal sequence of positions and related paths, and tracking interactions between cows [21,22,23,24]. Since in the literature it was found that heat stress influences cow behaviour [25,26], the application of these monitoring systems could be helpful in detecting and mitigating heat stress events as was previously done by Tsai et al. [27].

The main limitation of these monitoring systems is the reduced number of behaviours that can be detected. In detail, collar sensors and leg sensors are able to monitor feeding and ruminating, and lying and standing, respectively. To increase information on animal behaviour and overcome this limitation, a combination of different systems is applied in both indoor (i.e., dairy houses, cattle houses) [21,28] and outdoor (i.e., grazing) [29,30]. One of the positive aspects is that the identification of the animal’s position by a combination of sensors could provide hints about animal behaviours. For example, if the cow is located in the resting area, the feeding behaviour is not possible. Based on this detailed information, many studies combine sensors to permit a more reliable and accurate indication of animal welfare and health [28,31].

Recently, ultra-wideband (UWB) technology has been applied for tracking animals. This wireless technology is based on the transfer of data at high rates for short distances and low power densities [31]. UWB-based systems generally consist of hardware components (i.e., multiple anchors and tags) and software. Few studies have been carried out on UWB in dairy systems. In detail, Pastell et al. [18] developed a filter for positioning data in a free-stall dairy barn. Hindermann et al. [32] implemented a real time location system by using a commercially available UWB chip. Benaissa et al. [28] combined accelerometers and UWB systems to detect calving and oestrus events. The presence of animals or equipment in the barn can interfere with the UWB tracking system or hide the signal effects on the data acquired. In this context, UWB calibration and validation represent an important step for monitoring even though guidelines are not available [33,34,35]. Although this UWB system is generally applied in field experiments, in the literature few studies have been carried out on validation of the instruments. Benaissa et al. [28] carried out a static validation of the UWB location system to assess the accuracy and precision in stationary scenarios. Porto et al. [36] evaluated the UWB performance for localisation and identification of animals by previously calibrating instruments in situ. Melzer et al. [33] assessed the influence of system calibration, and data filtering and smoothing methods on tracking data in a dairy barn by UWB. These studies mainly focused on the validation of sensors in field conditions. There is a lack of information in the validation of sensors at the laboratory scale. 

Challenges still exist in assessing the adequacy of the available systems to monitor individual animals in specific environments, and in the design of new systems. Therefore, the main purpose of this research was to evaluate the performance of the SEWIO ultrawide-band (UWB) real time location system (RTLS) for the identification and localisation of cows during their activity in the barn through preliminary analyses in laboratory conditions. The objectives included the quantification of the errors performed by the system in laboratory conditions, and the assessment of the suitability of the system for real time monitoring of cows in dairy barns.

## 2. Materials and Methods

### 2.1. SEWIO UWB System and Experimental Setting

**Figure 1:**
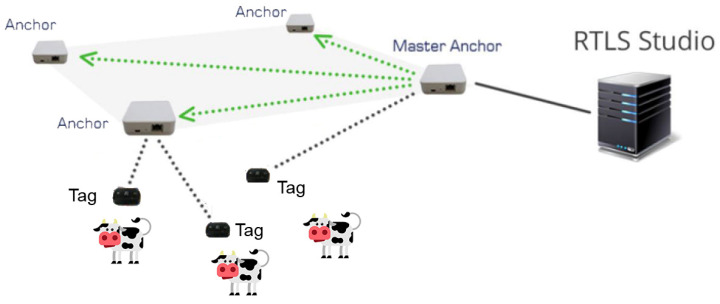
Scheme of the SEWIO UWB system. The black dotted lines and the green dotted lines represent the Blink layer and the Sync layer, respectively. Source: Adapted from [37].

Measurements were carried out in a laboratory (13.8 m × 5.40 m) by the use of a real time UWB-based location system (SEWIO, Brno, Czech Republic). The SEWIO UWB system consists of anchors, tags and the software RTLS Studio (Figure 1). The SEWIO system is a UWB technology based on the time difference of arrival. The SEWIO system has two types of UWB communication based on two layers. The first layer is named the blink layer where the communication occurs between the anchors and tags. The tags send UWB messages (i.e., blinks) to the anchors; then, the anchors receive these UWB blink messages, process the received information, and send information to the RTLS server. Finally, RTLS Server computes the precise position by using the UWB blink message. 

The second communication layer is named the Sync layer based on the communication between the anchors. To estimate the position with accuracy, the anchors need to be accurately synchronized by using a master anchor within the system. The server periodically sends information to these master anchors to synchronize neighboring anchors. Then, the master anchors send synchronization messages via UWB to the slave anchors in their vicinity.

In this study, six anchors (i.e., A, B, C, D, E, F) were located at 3 m height from the floor, one of them was the master (i.e., A). The position of the master anchor was considered the origin of a Cartesian coordinate system based on a preliminary test suggested in the SEWIO guide [37]. The calibration was carried out by using software provided by SEWIO. 

**Figure 2:**
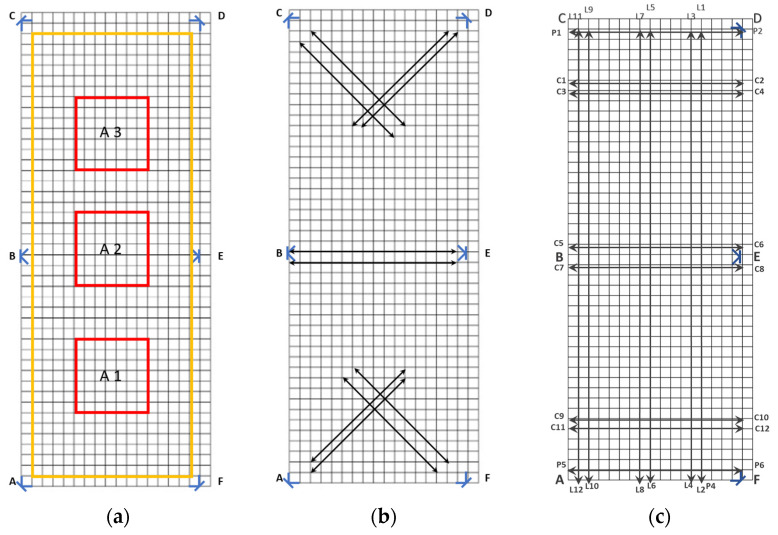
Position of the tag during the three experimental trials: (**a**) localisation at different areas (i.e., central areas of the rectangle A2, central area between 4 anchors, A1 and A3; perimeter of the laboratory); (**b**) approaching and moving from an anchor; (**c**) dynamic paths at 0.2 m s^−1^ and 1 m s^−1^.

Data were acquired by locating a tag in specific positions in the laboratory and all the anchors were used to calculate the tag position. The origin of the coordinate system was fixed at the bottom left corner of the investigated area, i.e., in point A of Figure 2. The x and y coordinates related to the position of the tag (i.e., real position) as well as the x and y coordinates detected by the UWB system were recorded in a dataset by a skilled operator. 

### 2.2. Study Plan

Different experimental trials were carried out to acquire data by simulating the position and movement of the cows (Figure 1). In the first experiment (Figure 2a), the x and y coordinates of points located in the central areas (i.e., A1, A2 and A3) and on the perimeter of the laboratory were acquired. In the second experiment (Figure 2b), the x and y coordinates of points approaching and moving from anchors were recorded. In the third experiment (Figure 2c), the x and y coordinates of points at different tag velocities were acquired. In detail, dynamic points were acquired at dynamic paths of 0.2 m s^−1^ and 1 m s^−1^ to simulate slower and faster movement of the animals along the service and feeding alleys [8]. The dynamic paths are the longitudinal and transversal paths shown in Figure 2c.

### 2.3. Statistical Analyses

One-way and two-way analysis of variance (ANOVA) was applied to test the differences between groups of parameters (i.e., based on the position and movement of the tag) for errors related to the x and y coordinates (i.e., E_x_ and E_y_) and accuracy. The error related to the x and y coordinates was assessed as the difference between the x coordinate (or y coordinate) measured by the UWB and the x coordinate (or y coordinate) of the reference system. The accuracy was determined as the mean Euclidean distance between the position recorded by the UWB system and its real position as it was recently determined by Benaissa et al. [28]. The level of significance applied in this study is considered a *p* value lower than 0.05. When the one-way ANOVA was significant, the Tukey test post hoc analysis was carried out to compare different groups. The groups of E_x_, E_y_ and accuracy analysed were the following: data acquired in the center of the study area and in the perimeter of the investigated area; different groups at the center of the study area (i.e., A1, A2, A3); groups of different approaching and moving paths from anchors (i.e., A, B, C, D, E, F) in the experimental laboratory; different tag velocities (i.e., 0.2 m s^−1^ and 1.0 m s^−1^) for transversal and longitudinal paths. Moreover, correlation and regression analysis were carried out. Correlation quantifies the relationship between variables whereas regression gives information on how the change in one variable will affect another variable. 

## 3. Results 

### 3.1. First Experiment

Under static conditions the errors E_x_ and E_y_ ranged from −1.37 m to 1.05 m, and from −1.20 m to 1.48 m, respectively. The results of the linear regression model highlighted a significant relationship between the measured coordinates and real coordinates under static conditions with a *p* value below 0.001 for both x and y coordinates. The regression equation for the x coordinate is x_measured_ = 0.51 + 0.71 x_real_ with the adjusted coefficient of determination R^2^_adj_ equals 96.2%. The regression equation for the y coordinate is y_measured_ = 0.59 + 0.92 y_real_ with the adjusted coefficient of determination R^2^_adj_ equal to 99.8%. 

Results related to the first experiments are shown in Table 1. The results of the one-way ANOVA showed that there was a significant difference (*p* < 0.001) between error related to x and y coordinates in the central area and perimeter of the investigated laboratory. In detail, the mean values of E_x_ and E_y_ in the central area were higher than those in the perimeter (Table 1). The E_x_ ranged from −1.34 m to 0.51 m and from −1.37 m to 1.05 m in the central area and on the perimeter, respectively. The E_y_ ranged from −0.75 m to 0.49 m and from −1.20 m to 1.48 m in the central area and on the perimeter, respectively. The E_x_ and E_y_ affect the accuracy of the measurement. In fact, accuracy for points measured on the perimeter was significantly (*p* < 0.001) lower than that for points measured in the center of the barn. The accuracy ranged from 0.02 m to 1.36 m and from 0.10 m to 1.66 m in the central area and on the perimeter, respectively.

Generally, measurements with UWB overestimated the position with the exception of the Ex in the central area that underestimated the measurement. When comparison was carried out for points located in different central areas in the laboratory, higher errors and lower accuracy were detected far from the master anchor.

### 3.2. Second Experiment

**Figure 3:**
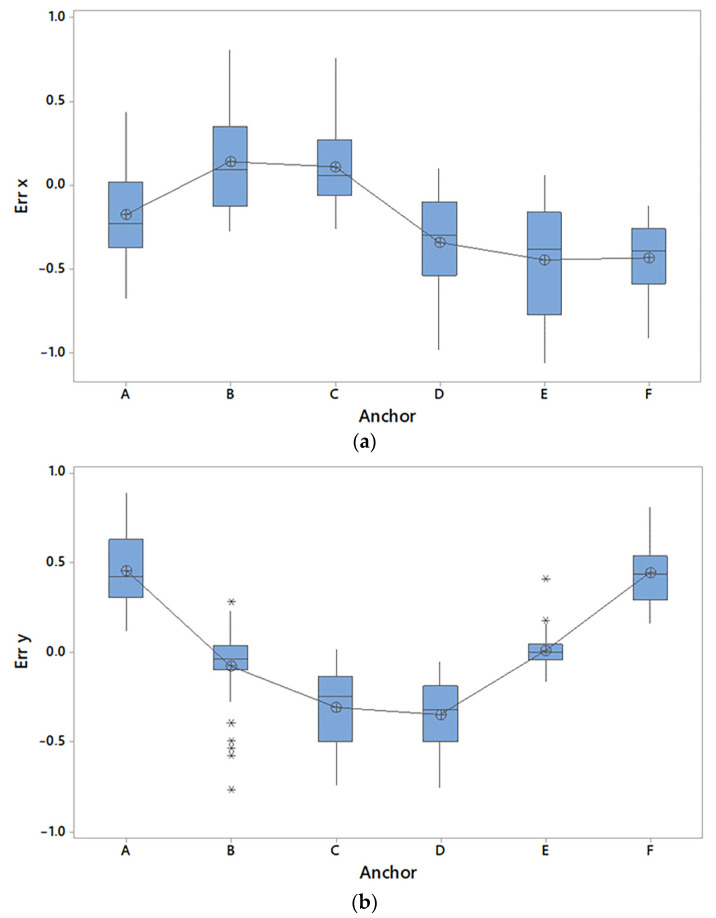
Boxplot of the E_x_ (**a**) and E_y_ (**b**) measured when tag was approached or was moved from a specific anchor (i.e., A, B, C, D, E, F).

Based on the two-way ANOVA, errors E_x_ and E_y_ were not influenced (*p* > 0.6) by the movement of the tag (i.e., approaching and moving from anchors) and the interactions between the movement of the tag and the position of the anchors. Results (Table 2) related to E_x_ and E_y_ coordinates of investigated points approaching and moving from anchors showed that the errors were influenced only by the position of the anchors in both x and y directions (*p* < 0.001). The accuracy related to anchors F and A was the lowest and related to anchors C and B were the highest. Based on Figure 3, the boxplot showed that E_x_ was overestimated in anchors B and C, whereas the measurements were underestimated in anchors A, D, E and F. The highest E_y_ (Figure 3b) was recorded in anchors A and F, whereas the lowest E_y_ was recorded in anchors C and D. 

**Figure 4:**
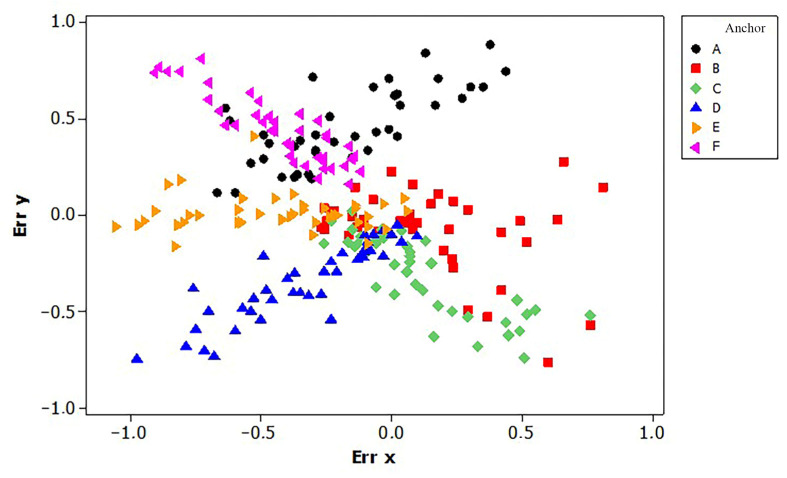
Scatterplot of the error along the x (**Err x**) and y (**Err y**) axes.

In detail, the scatterplot in Figure 4 shows the distribution of the E_x_ and E_y_ when a tag was approached or was moved from a specific anchor. Based on the correlation analysis, E_x_ and E_y_ had a great correlation for anchors A (r = 0.73 and *p* < 0.001), C (r = −0.82 and *p* < 0.001), D (r = 0.89 and *p* < 0.001), F (r = −0.89 and *p* < 0.001). It was found that Ex and Ey are directly proportional in anchors A and D, whereas E_x_ and E_y_ are inversely proportional in anchors F and C. The regression analyses showed R-adj^2^ higher than 70% only for anchors D and F with R-adj^2^ equal to 78.6% and 78.1%, respectively. The anchor E showed error only along the x axis and, consequently, there was not a significant correlation between E_x_ and E_y_ in E with *p* = 0.9. The errors related to B showed higher distribution compared to those acquired in other anchors and, thus, results in Figure 4 showed a deviation of the path. This is related to the position of the anchors on plan distribution. For this reason, it is of utmost importance to an accurate position of the anchors because slight differences in the installation process (i.e., tilt angle) could increase errors. 

### 3.3. Third Experiment

**Figure 5:**
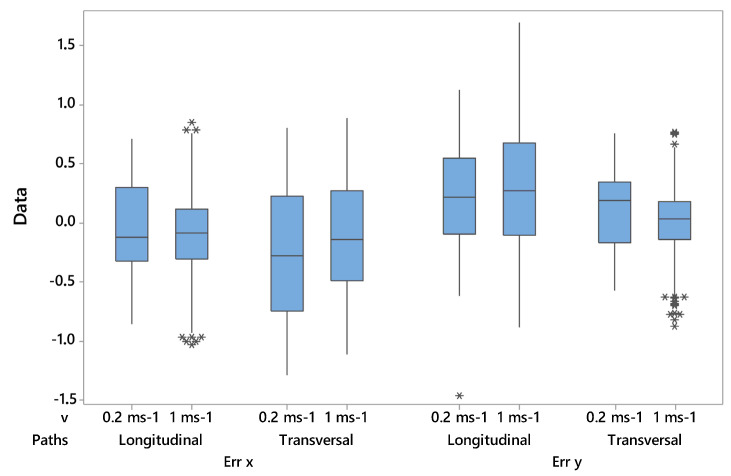
Boxplot of E_x_ and E_y_ at different groups of velocities (i.e., 0.2 ms^−1^ and 1 ms^−1^) and paths (i.e., longitudinal and transversal).

Under dynamic conditions, the errors E_x_ and E_y_ ranged from −1.29 m and 0.89 m, and −1.47 and 2.81, respectively. The results of the linear regression describe a significant relationship between the x (or y) measured coordinate and the x (or y) real coordinate under dynamic conditions with *p* < 0.001 for both coordinates. The regression equation for the x coordinate is x_measured_ = 0.31 + 0.82 x_real_ with the adjusted coefficient of determination R^2^_adj_ equals 98.0%. The regression equation for the y coordinate is y_measured_ = 0.22 + 0.99 y_real_ with the adjusted coefficient of determination R^2^_adj_ equals 99.5%. The simulation on dynamic movements (Table 3) showed that there was not a significant difference (*p* > 0.05) between E_x_ (or E_y_) at different tag velocities for longitudinal paths, whereas there was a significant difference (*p* < 0.001) between E_x_ (or E_y_) at different tag velocities for transversal paths. In detail, higher errors were recorded when the tag was moved at 0.2 m s^−1^. The boxplot in Figure 5 shows that E_x_ and E_y_ for velocities at 0.2 m s^−1^ in transversal paths had the widest range. With regard to accuracy, a significant difference was found between different tag velocities for both longitudinal and transversal paths (*p* < 0.001). The highest accuracy was found when a tag was moved at 0.2 m s^−1^ in longitudinal paths and at 1.0 m s^−1^ in vertical paths. 

**Figure 6:**
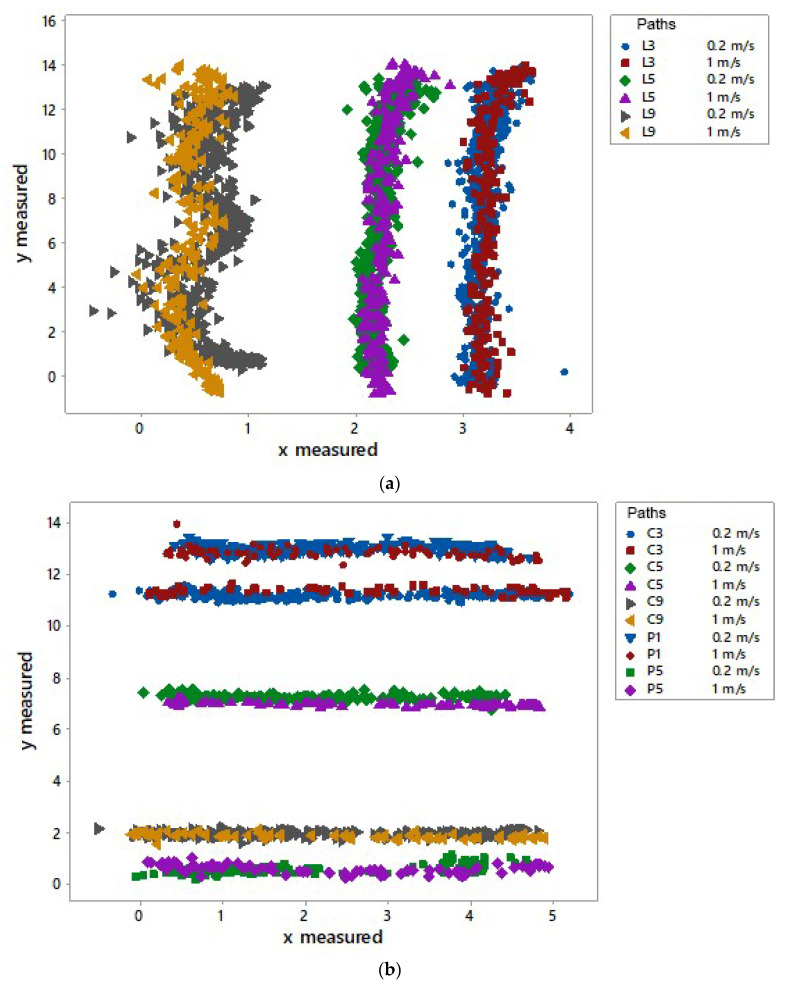
Scatterplot of the coordinates recorded by UWB system in specific longitudinal (**a**) and transversal (**b**) tag’s paths.

Figure 6 shows the scatterplot of coordinates measured by the UWB system in specific longitudinal and transversal paths analysed in this study. The results showed that the UWB system is able to provide the specific paths of the tag. Close to anchor B, higher errors were found in the previous error analysis with effects on the representation of the tag’s path. These effects are evident along the longitudinal paths close to the anchor B (Figure 6a) where it was recorded a deviation of the path. In transversal paths (Figure 6b), the x coordinates measured by the UWB system were shifted from the origin of the axis. 

## 4. Discussion

### 4.1. Performance of the SEWIO UWB RTLS

Based on the results, a different position in the investigated area produced an error in both directions of the coordinate system (i.e., E_x_ and E_y_). The ununiform error related to a different tag position was found in the study of Hinderman et al. [32], where E_x_ and E_y_ in the central area were higher than those in the perimeter with effects on the accuracy. Overall, the Sewio UWB system had an accuracy of 0.5 m, in line with the previous study of Porto et al. [36] carried out on a different UWB system. The authors used a Real-Time Location System (RTLS) based on Ubisense UWB technology within a semi-open free-stall barn. The UWB Ubisense system had an accuracy of 0.51 m. The UWB system analysed by Benaissa et al. [28] had a greater accuracy of 0.21 m. A similar result was found by Melzer et al. [33] that obtained an accuracy of 0.20 m by increasing the number of anchors (i.e., fourteen anchors) in an area characterised by similar dimensions compared to those reported in the study of Benaissa et al. [28] (i.e., six anchors). In the study by Frondelius et al. [38], the distance between the sample point and the measuring point was 0.17 ± 0.17 m in single measurement point tests using the Ubisense system. In this system, the error increased significantly when the tags were attached to cow’s collars in a full barn compared to measurements made with just the tags in the same barn while it was empty. The Ubisense system was also applied in the study of Pastell et al. [18], where a filtering method was applied.

In the study of Chapa et al. [22], the Smartbow RTLS was used to detect localisation of cows in alleys, feed bunks, and cubicles. The system had an overall accuracy of 87.6%. The results of Wolfger et al. [19] showed a mean distance difference of 1.22 m for dynamic measurements.

Based on the results obtained with the SEWIO UWB system, when comparison was carried out among points located in different central areas in the laboratory, higher errors and low accuracy were detected far from the master anchor. The results of this study showed that the position of the anchors in both x and y directions can influence the accuracy of the measurement. The experiments in this study also demonstrate that the system has a greater error in the edges of the measurement area. It could be beneficial to place the anchors outside of the area which animals can access. In the literature, the accuracy could be improved by using a great number of anchors. In fact, Meunier et al. [34] improved the accuracy of the system by increasing the number of anchors compared to other studies above mentioned. Increasing the number of anchors could be a great solution for data quality, especially in small areas such as concentrate feeders and drinking areas where the system performance could be negatively affected by the reduced dimensions. This solution could be useful for research purposes, by could be expensive for farmers [28]. However, increasing the number of anchors in the feeding and lying areas could improve data accuracy in view of optimising herd management. Moreover, it could be beneficial to add stationary tags in both good and potentially problematic areas in field conditions to improve the accuracy of the measurement [33]. 

In some studies, filtering and smoothing methods have been successfully applied, obtaining an increase in accuracy [33,34]. As it was reported by Pastell and Frondelius [18], the use of filtering makes it possible to reduce the position error of the system below 1 m. The exception is represented by the corner points where the error could reach 2 m. However, the use of filtering and smoothing methods requires more computational power and may cause a delay in the case of real-time monitoring [28]. Another relevant aspect is that under field conditions, the UWB system should be combined with other systems. The time that cows spend at a specific location (e.g., alley, cubicles, and feed bunk) does not mean that the animal is actually involved in that activity (i.e., walking, lying, and feeding). For instance, if a cow is in the resting area it does not ensure that the animal is in lying, especially under stress conditions.

In this study, the error was ununiform along the x and the y axes where the highest E_y_ was recorded in anchors A and F, whereas the lowest E_y_ was recorded in anchors C and D. An ununiform error distribution was found in the study of Benaissa et al. [28] that found higher errors along the y axis than those along x axis in field conditions. 

Although the SEWIO UWB system is able to represent the path of a specific tag, the position of the anchors on plan distribution can produce effects on the error with a deviation of the path (as it was found in the longitudinal axis) and/or a shift of the coordinates (i.e., as it was found in the transversal paths). Similar results were found in the study of Hidermann et al. [32] where errors along the x axis were more evident on the right side of the barn than those on the left side. It is of utmost importance to have an accurate position of the anchors because slight differences in the installation process (i.e., tilt angle) could increase errors. Moreover, the accuracy was lower in specific areas of the barn due to the interaction of the signal with materials of the barn [18,22,36] and water bodies [21]. In fact, Benaissa et al. [28] assessed the accuracy of the UWB system in stationary scenarios and found that the accuracy was lower close to the milking robot due to the presence of a concrete ceiling that produces multipath effects as well as extra signal losses.

Furthermore, difficulties in tracking cows under field conditions are related to the features of tags located on the animals, such as being ergonomic and protected from water and dust [14].

### 4.2. Applicability of the SEWIO UWB RTLS and Further Perspectives

In this study, the experiments were carried out in a laboratory that simulated the pen dimensions. The UWB system was preliminarily tested at the laboratory scale to assess the performance of the system when the influencing factors of the barn environment are excluded. The errors analysis of this study has shown that UWB could be applied for cow tracking since errors were compatible with the cow dimensions. According to Huhtala et al. [39], the accuracy should be lower than 1 m for tracking cows and in this study, the accuracy was equal to 0.50 m under laboratory conditions. However, validation under field conditions could add more information about the reliability of the monitoring system. In detail, it would be interesting to compare the laboratory results with the errors of the SEWIO UWB system evaluated in field conditions in a dairy barn to define whether and to what extent errors increase in tracking animals. The farm structure and the presence of the animals could affect the performance of the UWB system and its applicability in livestock buildings due to many factors.

The first one is the building materials of the livestock buildings. Nowadays, dairy barns are mainly built in steel and concrete, and those materials can affect the accuracy of the measure. In the study of Meunier et al. [34], it was found an improved accuracy of 0.16 m by excluding the milking robot area where many parts are composed of metals. Under field conditions, the presence of steel beams, pillars, nets for birds, feed bunker, and separators for stalls and alleys is very common in dairy barns and the performance of the SEWIO RTLS could be influenced by the interaction between the signal and these steel elements.

The second aspect is the presence of many animals in the dairy barn that can affect the signal [18]. Although this aspect could be the source of errors due to the body mass of the cows, the SEWIO UWB is able to monitor many animals in the barn at a time. Deep knowledge of the SEWIO UWB under real conditions could allow analysing of the system performance in the monitoring of the interaction between different cows.

Moreover, a further evaluation should consider a dairy barn equipped with sprinklers or fogging systems, especially in Mediterranean areas where these systems provide cooling to the animals to reduce their heat stress [40,41]. The presence of the cooling system may increase the negative effects on the accuracy of the measurements. An accurate system, especially in the Mediterranean area, could be helpful in monitoring heat stress during warm periods and consequently improve the management of the barn.

In this study, the SEWIO UWB application in a dairy pen was simulated by considering similar dimensions in real conditions. The working conditions of the SEWIO UWB require respecting specific suggestions in the localisation of the anchors and tags (i.e., try to keep a direct line of sight between the tag and the anchors, maintain the ratio between the two sides of the investigated area lower than 3:1). Therefore, the localisation of these components in the barn could be subjected to modifications due to difficulties encountered in the installation process. 

The above-mentioned factors have implications for the applicability of the SEWIO UWB system in real-world scenarios. It is expected that the accuracy will be lower under field conditions compared to that found in this study and further research is needed to quantify how much these factors can affect the performance of the SEWIO UWB. 

## 5. Conclusions

This research study assessed the use of real time monitoring based on the SEWIO UWB technology under laboratory conditions. By computing errors and accuracy, the UWB system has errors and accuracy similar to other studies found in the literature and could be applied in livestock buildings to assess the performance of this monitoring system to track animal behaviour. In detail, it should be possible to simulate under laboratory conditions the position of the animals or their path in both longitudinal and vertical paths. The use of SEWIO UWB system could be useful to locate animal position in the barn as well as to measure time animals spend in specific area in the barn. Further studies in field conditions are necessary to identify final recommendations for the use of this technology in dairy barns. Since it was found that the position of the anchors could influence errors and accuracy, further studies are required to determine correction coefficients to reduce the effects of the anchors’ position on data analysed as well as improve data quality in field experiments. In these conditions, data could be influenced by the presence of different materials in the barn where cows are monitored. Further studies are necessary to investigate and compare data between laboratory and in-filed conditions.

## Figures and Tables

**Table 1:** Results of the one-way ANOVA related to errors and accuracy at different locations in the experimental laboratory. E_x_, E_y_ and accuracy are expressed in meters.

Comparison	Tag Position	E_x_	SD E_x_	E_y_	SD E_y_	Accuracy	SD
Errors between tag located in the centre vs. perimeter of the barn	Central area	−0.23 ^a^	0.2	0.22 ^a^	0.15	0.31 ^b^	0.21
	Perimeter	0.05 ^b^	0.59	0.06 ^b^	0.65	0.82 ^a^	0.39
Errors in different areas at the centre of the laboratory	A1	−0.32 ^c^	0.16	0.24 ^a^	0.08	0.42 ^a^	0.13
	A2	−0.23 ^b^	0.23	0.03 ^b^	0.11	0.27 ^b^	0.20
	A3	−0.15 ^a^	0.16	−0.07 ^c^	0.08	0.20 ^c^	0.13

^a–c^ Groups of values that do not share a letter (a, b, c) are significantly different, within each column.

**Table 2:** Results of the one-way ANOVA related to errors (i.e., E_x_ and E_y_) and accuracy at different approaching and moving paths from anchors in the experimental laboratory. E_x_, E_y_ and accuracy are expressed in meters.

Tag Position	E_x_	SD E_x_	E_y_	SD E_y_	Accuracy	SD
A	−0.17 ^b^	0.30	0.46 ^a^	0.20	0.58 ^a^	0.16
B	0.14 ^a^	0.31	0.03 ^b^	0.11	0.32 ^c^	0.24
C	0.11 ^a^	0.25	−0.30 ^c^	0.21	0.38 ^bc^	0.25
D	−0.34 ^bc^	0.28	−0.34 ^c^	0.20	0.49 ^ab^	0.32
E	−0.44 ^c^	0.32	0.01 ^b^	0.10	0.46 ^abc^	0.31
F	−0.43 ^c^	0.22	0.44 ^a^	0.18	0.62 ^a^	0.27

^a–c^ Groups of values that do not share a letter (a, b, c) are significantly different, within each comparison and a specific coordinate.

**Table 3:** Results of the one-way ANOVA related to errors at different approaching and moving paths from anchors in the experimental trials carried out in laboratory.

Paths	Tag Velocity	E_x_	SD E_x_	E_y_	SD E_y_	Accuracy	SD
Longitudinal paths	0.2 m s^−1^	−0.02 ^a^	0.36	0.23 ^a^	0.43	0.53 ^b^	0.28
	1.0 m s^−1^	−0.04 ^a^	0.38	0.28 ^a^	0.56	0.64 ^a^	0.34
Transversal paths	0.2 m s^−1^	−0.24 ^b^	0.54	0.11 ^a^	0.30	0.61 ^a^	0.29
	1.0 m s^−1^	−0.13 ^a^	0.46	0.02 ^b^	0.28	0.50 ^b^	0.25

^a,b^ Groups of values that do not share a letter (a, b) are significantly different, within each path and a specific coordinate.

## Data Availability

Data are available on request.
